# Evaluation of Soft and Hard Tissue Changes Following the Use of Rapid Molar Intruder for the Management of Skeletal Anterior Open Bite in Mixed Dentition: A Randomized Controlled Trial

**DOI:** 10.7759/cureus.32826

**Published:** 2022-12-22

**Authors:** Amjad Ali Hasan, Hammam M Zeidan, Mohammad Y Hajeer, Azzam Al-Jundi, Rabab Al-Sabbagh, Tarek Z. Khattab, Mohamad Kusai Al-Munajed

**Affiliations:** 1 Department of Orthodontics, University of Damascus Faculty of Dentistry, Damascus, SYR; 2 Department of Orthodontics, University of Al-Baath Faculty of Dentistry, Homs, SYR; 3 Department of Orthodontics, University of Hamah Faculty of Dentistry, Hamah, SYR

**Keywords:** correction of the open bite, vertical occlusal correction, untreated control group, soft-tissue changes, cephalometric changes, skeletal anterior open bite, skeletal open bite, rapid molar intruder, mixed dentition, anterior open bite

## Abstract

Objective

The aim of the study was to evaluate skeletal, dentoalveolar, and soft tissue changes following early anterior open bite (AOB) treatment using a rapid molar intruder (RMI).

Materials and methods

A two-arm, parallel-group, randomized controlled trial was conducted on 40 patients aged 8-12 years with anterior open bites. They were randomly allocated to the RMI group and the untreated control group (UCG) with a 1:1 allocation ratio. At the beginning of the treatment (T1) and after nine months of treatment (T2), lateral cephalometric images were taken of each patient. The primary outcome measures were skeletal, dentoalveolar, and soft tissue changes. A two-sample t-test was used in the intergroup comparisons of the cephalometric measurement.

Results

The findings showed that the overbite increased significantly in the RMI group compared to the control group (*x *= 4.44 mm, *x *= 0.19 mm, respectively; p<0.001). A statistically significant intrusion of the upper and lower first molars was observed in the RMI group (*x *= 2.9 mm, *x *= 1.54 mm, respectively) compared to a slight extrusion in the control group. The differences between the two groups were significant (p<0.001). The SN: GoMe angle and the sum of Bjork decreased significantly in the RMI group compared to an increase observed in the control group. The differences between the two groups were significant (p<0.001).

Conclusion

The rapid molar intruder is an effective appliance for correcting anterior open bites in mixed dentition, inducing favorable skeletal, dentoalveolar, and soft tissue changes.

## Introduction

The skeletal anterior open bite (AOB) presents a major challenge in orthodontic treatment [1] due to certain factors, such as facial esthetics and the incidence of relapse, which make it one of the most difficult cases to be treated by orthodontists [2]. The benefits of the early treatment of the skeletal AOB can be noticed by avoiding the risks of late orthodontics surgical treatment and improving the child’s self-confidence [3].

Several therapeutic methods for correcting AOB have been suggested ranging from appliances that control bad oral habits (e.g., removable or fixed palatal cribs or spurs) [3] to functional appliances that modify the facial growth pattern (e.g., the open-bite Bionator, Frankel IV [4]). In addition, other appliances have been proposed to control the vertical dimension, such as fixed or removable posterior bite blocks [5], removable posterior bite blocks with magnets [6], springs [7], high-pull headgear, and the vertical chin cup [8].

Despite the available evidence of the benefits of these appliances, several disadvantages or shortcomings have been encountered. The results of the palatal crib may be confined to anterior dentoalveolar effects (extrusion of maxillary and mandibular incisors) [9], whereas the functional appliances (e.g., the open-bite Bionator, Frankel IV), the removable appliances, and the extraoral appliances (e.g., the high-pull headgear and vertical chin cup) require great patient compliance, which is not available in many cases [4]. In addition, the problems with the extraoral appliances are the difficulty in patient adaptation to them, their bulkiness, and their unsightly appearance, which limit the patient's cooperation. Magnets might lead to lateral crossbites [6] and springs tend to get broken during lengthy treatments [7].

It can be seen that most of the devices used in the previous studies have taken into account the patient’s cooperation. Thus, there is an urgent need for the application of vertical molar control or intruding it by a device that does not require the patient's cooperation. The rapid molar intruder (RMI) device was first suggested by Carano et al., who mentioned in two cases that the device would provide a strong solution for children with AOB [10]. Then, Çinsar et al., in a randomized controlled trial, showed the effectiveness of the RMI in treating anterior open bite in patients aged between 10 and 13 years [11]. However, the problems with these studies were the heterogeneity in the sample's age and the small sample size. Albogha et al. also used a fixed posterior bite block with an RMI for four months in their cohort study and found this treatment protocol effective for correcting both AOB and vertical dental imbalances in the mixed dentition. However, the study was a single-group short-term observational study without a control group to filter out the unknown confounding factors [6].

Furthermore, multiple systematic reviews have suggested the need to perform high-quality randomized controlled trials in this field [1,12-14]. Therefore, the current study aimed to evaluate skeletal, dentoalveolar, and soft tissue changes following the early treatment of AOB using a rapid molar intruder (RMI). The null hypothesis of this trial stated that there were no differences in the skeletal, dentoalveolar, and soft tissue changes between the RMI group and the untreated control group for patients with AOB in mixed dentition after nine months of active treatment.

## Materials and methods

Study design and settings

This study was a two-arm, parallel-group, randomized controlled trial. Ethical approval was obtained from the Local Ethics Committee of the Faculty of Dentistry, Al-Baath University (UBDS-2066-12032014PG/SRC4429). This trial was reported according to the Consolidated Standards of Reporting Trials (CONSORT) statement guidelines and was registered at ClinicalTrials.gov (ID: NCT05657522). Funding was received from the University of Al-Baath Postgraduate Research Budget (Ref no: 74692872301DEN).

Sample size calculation

The sample size was calculated using the G*Power 3.1.9.2 software (University of Kiel, Germany). The significance level was at 0.05, and the statistical power was at 90%. Based on the two-sample t-test, the effect size (1) of the overbite in Albogha et al. was used [6]. Therefore, the number of participants required in each group was 18. In the case of sample attrition, two patients were added to each group, with a total of 20 patients in each group.

Settings, participants, and eligibility criteria

**Figure 1:**
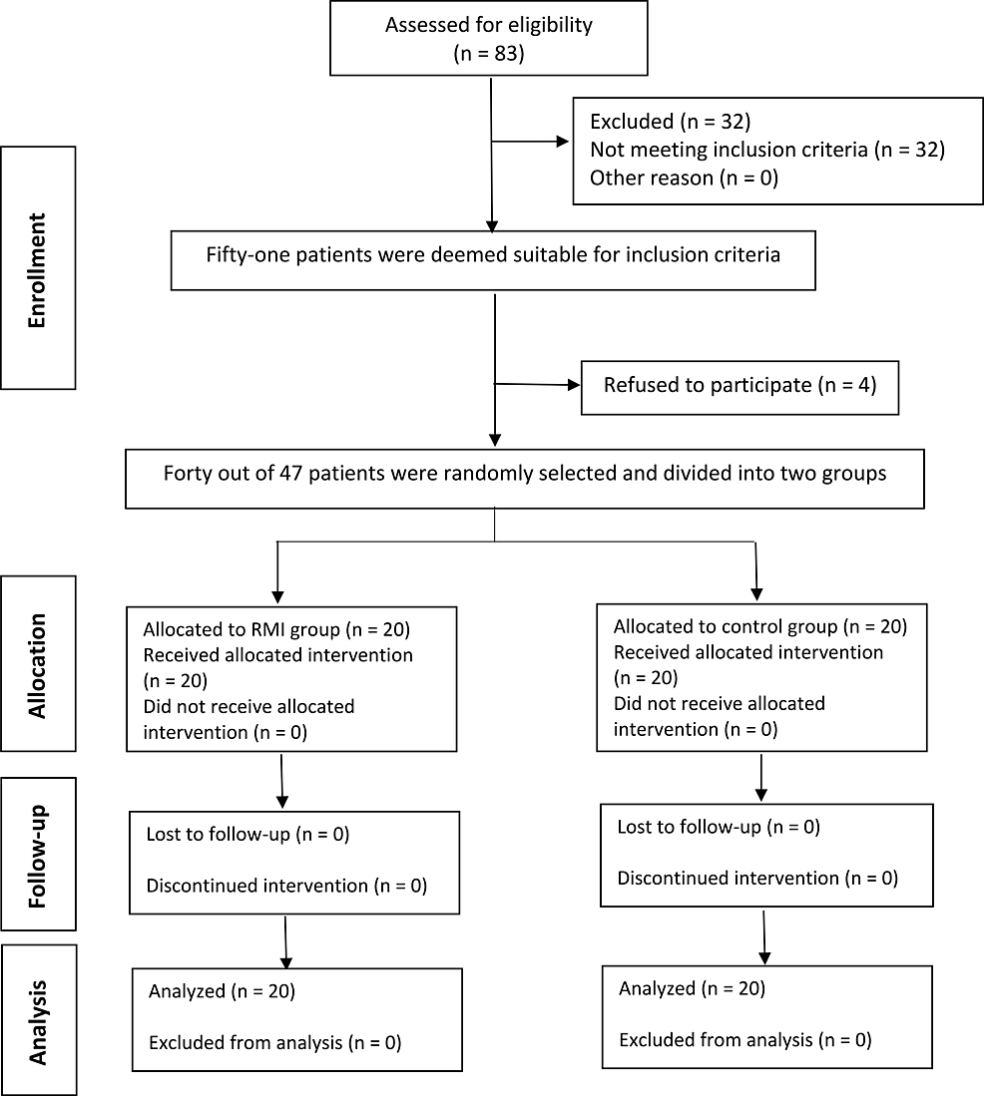
Consolidated Standards of Reporting Trials (CONSORT) flow diagram of patients' recruitment and follow-up

This trial was carried out at the Department of Orthodontics and Dentofacial Orthopedics, Faculty of Dentistry, University of Al-Baath, from September 2014 to April 2016. Thirteen primary schools were screened by (H.M.Z.) in Hama. Two thousand six hundred and thirteen students were examined; 83 patients were found with anterior open bites. Their families were invited to be treated at the Department of Orthodontics and Dentofacial Orthopedics, Faculty of Dentistry, Al-Baath University. Thirty-two patients did not meet the inclusion criteria, and 51 were found suitable for inclusion in this trial. Informed consent was obtained from parents or legal guardians of patients before trial commencement. However, four patients refused to participate in the experiment. Accordingly, 40 patients out of 47 were randomly selected, as presented in the Consolidated Standards of Reporting Trials (CONSORT) flow diagram (Figure 1).

The clinical inclusion criteria were AOB in the mixed dentition, chronological age between 8 and 12 years, Class I or II malocclusion, and good oral health. Exclusion criteria: previous orthodontic treatment, cleft lip/palate patients, craniofacial syndromes, obstruction in the nasal airway with persistent mouth breathing, and patients with finger-sucking habits. Patients with a mixed breathing pattern (i.e., mouth and nose breathers) were accepted in this trial. Also, those with secondary tongue thrust were included in this trial. The skeletal anterior open bite was assessed clinically and then confirmed radiographically using the following criteria: The angle between the mandibular plane and anterior cranial base (SN/GoMe) was greater than 33°, the intermaxillary angle (MM) was greater than 27°, and the facial axis (Y-axis) was greater than 65°.

Randomization and allocation concealment

Two study sample groups were randomly allocated: the rapid molar intruder (RMI) group and a control group of untreated patients. Simple randomization was used by an orthodontics specialist not involved in this research. Minitab® (Version 17; Minitab, LLC, State College, USA) was used for creating a randomization list with an allocation ratio of 1:1. Hidden information was sent through numbered and sealed envelopes for data collection. To maintain the sequence of allocation, the name, and date of birth of each participant were written on the envelopes. Then the data was transferred onto the allocation card inside each envelope. Upon completing the baseline assessment, corresponding envelopes were disclosed.

Rapid Molar Intruder (RMI) group

**Figure 2:**
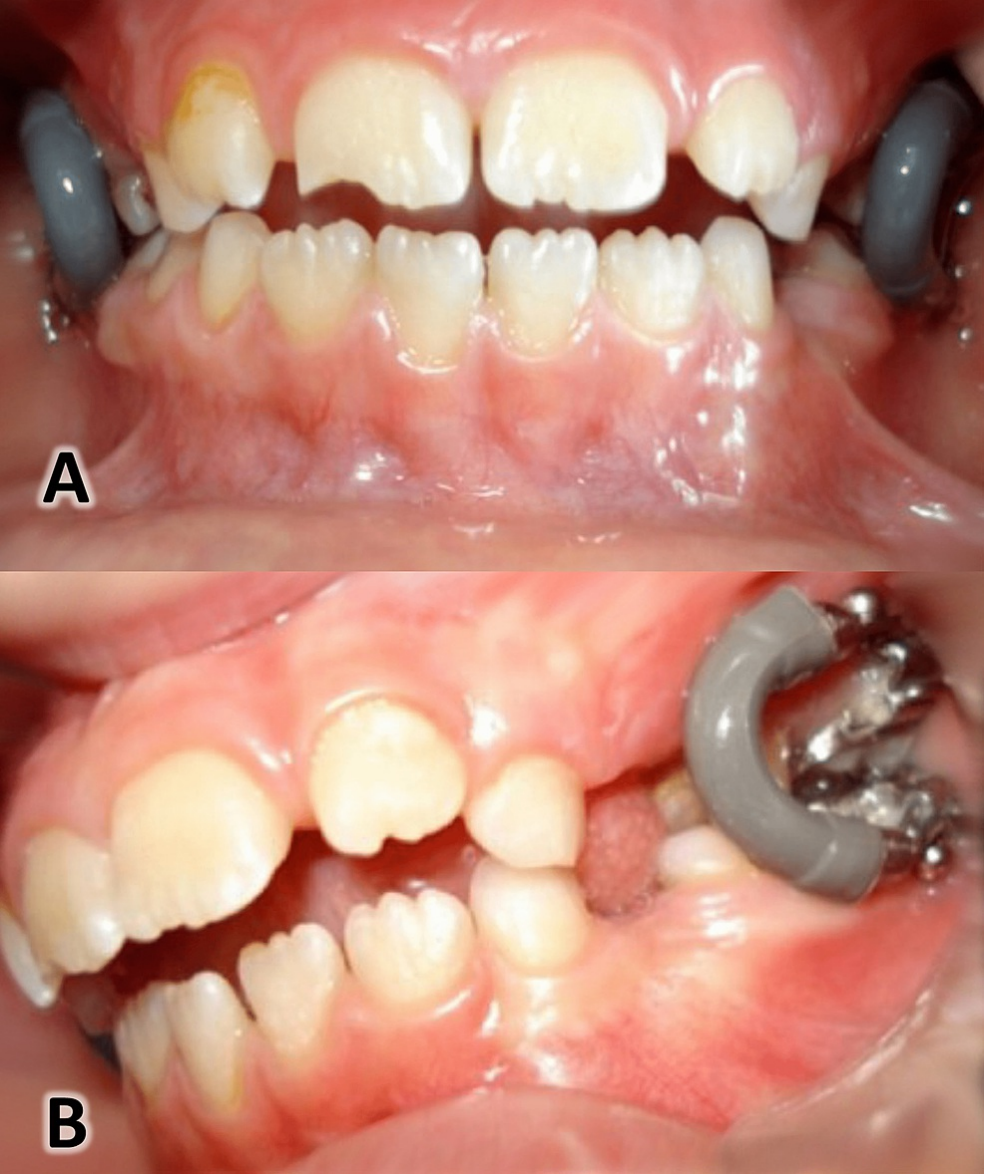
The rapid molar intruder (RMI). A: Frontal view. B: 45-degree view of the appliance in place.

In this group, patients were treated with the rapid molar intruder (RMI) by the principal researcher (H.M.Z.). The RMI consisted of the following components (Figure 2): bands on the upper first molars welded with a 0.9-mm-diameter round stainless steel transpalatal arch (TPA); bands on the lower first molars welded with a 0.9-mm-diameter round stainless steel lingual arch (American Orthodontics, Sheboygan, USA); and the main active element of nickel-titanium spring (RMI®, American Orthodontics, Sheboygan, USA) covered with a plastic material to protect the patient. These springs extend from the upper to the lower first molar bands with metal pins with a diameter of 0.060 inches to install the spring within a special tube on the molar bands. Bands were first cemented to the upper and lower first molars using glass ionomeric cement (Fuji I, Tokyo, Japan). The RMI active element was inserted the following day, i.e., 24 hours after the cementation of the bands. The RMI was used to intrude the upper and lower first molars together, and the TPA and lingual arch were used to prevent the molars from tilting to the buccal during the intruding process.

When the patient’s mouth is closed, the RMI device made an intruding force of about 800 grams on each side, and this force was expected to subside with time, becoming 450 grams at the end of the first week and 250 grams at the end of the second week, according to a preliminary report by Carano et al. [10].

A pedodontist (N.M.) at the Pedodontics Department at the University of Al-Baath Faculty of Dentistry performed a pulpotomy procedure of the upper and lower primary molars for each patient in the RMI group. Then an occlusal reduction of the posterior primary teeth was performed at the follow-up visits to allow for mandibular autorotation during the permanent molar intrusion. In each visit, the occlusion was examined, and the interocclusal distance between the upper and lower permanent first molars was measured. This distance was used to remove equal quantities from the primary molars' upper and lower occlusal surfaces using flat-end cylinder diamond burs (SO 21, Mani, Tochigi, Japan). This was done in each visit to reestablish the occlusal contact between the upper and lower permanent first molars. This procedure of occlusal reduction was repeated in each visit until the anterior open bite was corrected and a positive overbite was achieved. In the case of observing a tight contact between the upper and lower permanent first molars on both sides at the follow-up sessions, no reduction was performed. The protocol of occlusal reduction was accomplished by the principal researcher (H.M.Z.) and was supervised by one of the co-authors (M.K.A.).

The patients were asked to avoid wide mouth opening during talking or yawning due to the presence of the flexible spring between the two jaws. They were asked to keep their lips closed during rest positions to benefit from the provided treatment. The patients were followed up according to the following sequence: one week after the device's application, then every month for nine months. The objectives of the follow-up visits were: to check the safety and effectiveness of the device (the effectiveness of the flexible springs and the stability of the bands on the upper and lower first molars), to ensure that the TPA was not impinging upon the palatal mucosa; to ensure that the lingual arch was not impinging upon the lingual gingivae of the lower incisors; to reduce the occlusal surfaces of the upper and lower pulpotomized primary molars to allow for mandibular anterior rotation; to ensure that the patient adhered to the instructions; and to modify the device if necessary.

The untreated control group (UCG)

Without intervention, patients in the control group were monitored for nine months, and a lateral cephalometric image was taken. Following the observational period, patients were treated with the RMI or any alternative treatment modality used at the Department of Orthodontics.

Primary outcome measures: cephalometric variables

**Figure 3:**
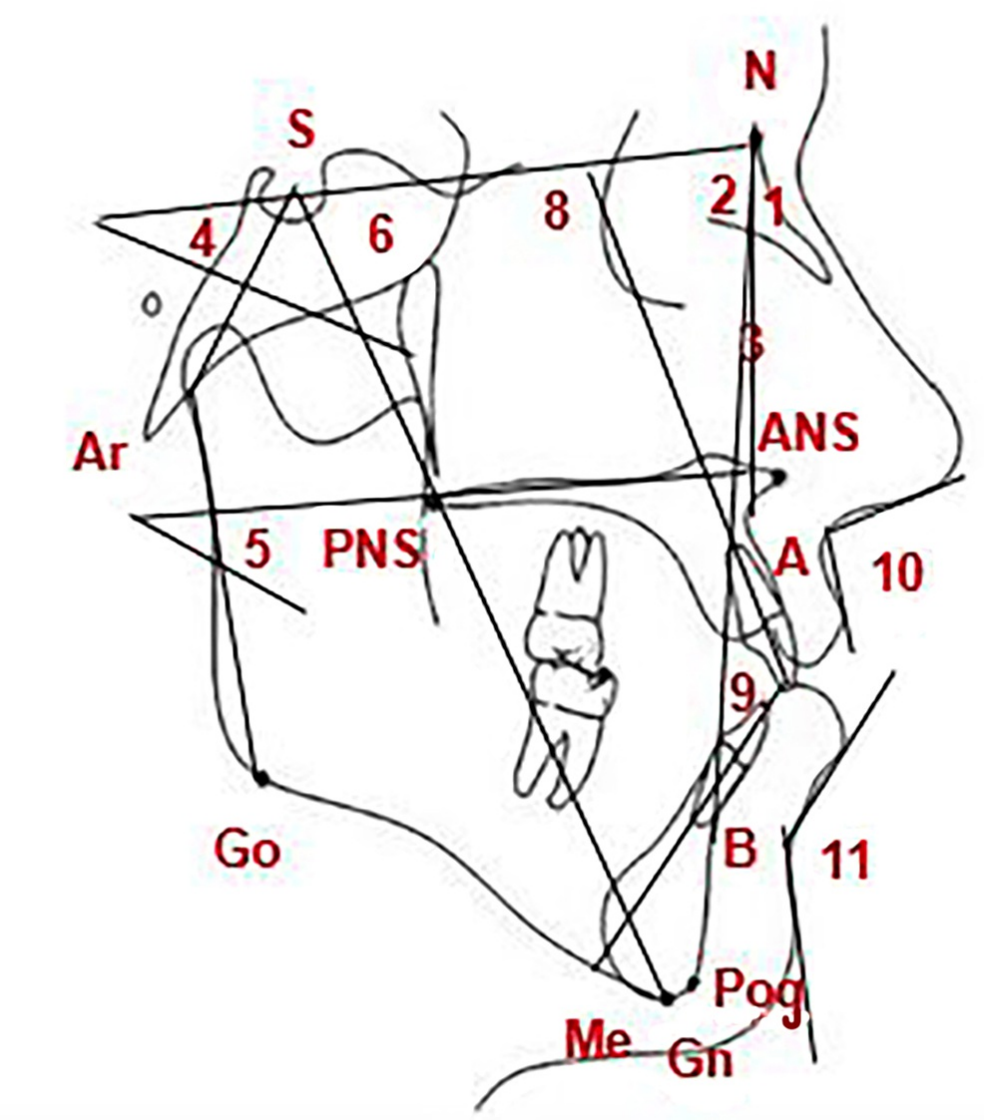
Angular measurement used in the cephalometric analysis 1, SNA; 2, SNB; 3, ANB; 4, SN.GoMe; 5, MM; 6, Y axis; 7, Björk Sum (N.S.Ar+S.Ar.Go+Ar.Go.Me); 8, U1.SN; 9, U1/L1; 10, Nasolabial; 11, Labiomental. N: most anterior and superior point on the nasofrontal suture; S: central point in the sella turcica; A: point of most concavity on the contour of the anterior upper jaw close to the root apices of the upper incisors; B: point of most concavity on the contour of the anterior lower jaw close to the root apices of the lower incisors; Pog: most prominent point on the anterior margin of the chin; Gn: the point that is most anterior and most inferior on the outer margin of the chin (symphysis); Me: lowermost point on the margin of the chin (symphysis); Go: the point located the angle of the mandible between the ramus and the body of the mandible; Ar: the point at the junction of the posterior border of the ramus and the inferior border of the body of the sphenoid bone; U1: upper incisors; L1: lower incisors; ANS: most anterior point on the maxillary bone (anterior nasal spine); PNS: most posterior point on the maxillary bone (posterior nasal spine); NS: the plane that connects points N and S.

**Figure 4:**
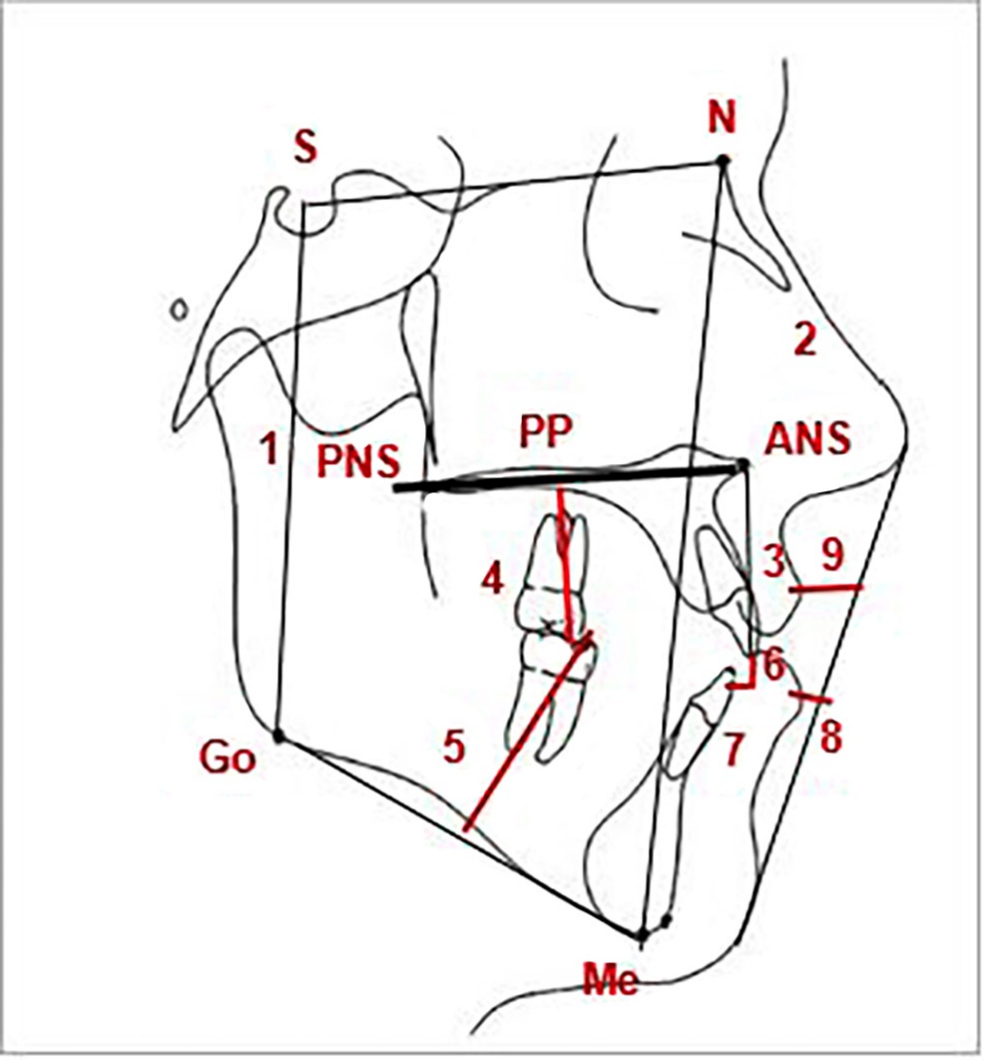
The linear measurement used in the cephalometric analysis 1, S-Go; 2, N-Me; 3, U1-PP; 4, U6-PP; 5, L6-GoMe; 6, Overbite; 7, Overjet; 8, E-Li; 9, E-Ls. S-Go: the plane that connects points S and Go; N-Me: the plane that connects points N and Me; PP: the plane that connects points ANS and PNS; U1-PP: upper anterior dentoalveolar height; U6-PP: upper posterior dentoalveolar height; L6-GoMe: lower posterior dentoalveolar height; Overbite: vertical overlap of the upper incisors; Overjet: horizontal overlap of the upper incisors; E-Li: distance between the Labrale inferius (Li) and E-Line of Ricketts; E-Ls: distance between the Labrale superius (Ls) and E-Line of Ricketts.

The skeletal, dentoalveolar, and soft tissue changes were the outcome measures. PaX‑i3D (VATEH Corporation, Ltd., Hwaseong, Korea) was used for lateral cephalometric acquisition with the same settings (at 50 kV, 4 mA, and 0.9-second exposure time). Focus-mid-sagittal-plane distance was fixed at 152 cm, and the film-mid-sagittal-plane distance was fixed at 16 cm. Each patient took lateral cephalometric images at the beginning of the treatment (T1) and after nine months of the first cephalograms (T2). Then Viewbox® (Version 4.0.0.98; dHAL Software, Kifissia, Greece) was used to evaluate 20 variables for each patient as defined by Jacobson [15] and Riolo et al. [16]. The definitions of these measurements are given in Table 1 and illustrated in Figures 3-4. Measurements were exported as Excel files (Office Excel 2019; Microsoft Corporation, Redmond, USA) and then analyzed statistically. The outcome assessment on cephalograms was performed by one of the co-authors (A.A.H.), who was completely blinded to which group the images belonged.

**Table 1:** Definitions of the angular and linear measurements These definitions are based on Jacobson [15] and Riolo et al. [16]

1	SNA	The angle between the SN plane and A point
2	SNB	The angle between the SN plane and B point
3	ANB	The angle between NA and NB lines
4	SN/GoMe	The angle between the SN plane and GoMe
5	MM	The angle between the palatal plane (PP) and GoMe
6	Björk Sum	The sum of N-S-Ar, S-Ar-Go, and Ar-Go-Me angles
7	Y-axis	The angle between the SN plane and Gnathion point
8	S-Go	The distance between S and Go
9	N-Me	The distance between N and Me
10	U1-PP (mm)	The upper anterior dentoalveolar height
11	U6-PP (mm)	The upper posterior dentoalveolar height
12	L6-MP (mm)	The lower posterior dentoalveolar height
13	U1/SN	The angle between the long axis of the upper incisor and the SN plane
14	U1/L1	The angle between the long axis of the upper and lower incisor
15	OB	The vertical overlap of the upper incisors
16	OJ	The horizontal overlap of the upper incisors
17	E-Li	The distance between the Labrale inferius (Li) and E-Line of Ricketts
18	E-Ls	The distance between the Labrale superius (Ls) and E-Line of Ricketts
19	Nasolabial angle	The angle between the nose and the upper lip
20	Labiomental angle	The angle between the chin and the lower lip

Assessing the error of the method

To assess the systematic and random error of the measurements, 20 lateral cephalometric images were randomly selected and reanalyzed two months after the first assessment by the same examiner (A.A.H.). Paired-sample t-tests were used to detect any possible systematic errors, whereas the intra-examiner reliability (random error) was determined using the intraclass correlation coefficients (ICCs).

Statistical analysis

Statistical analyses were performed using the SPSS program (version 22.00; IBM Corporation, Armonk, USA), and the significance level was set at 0.05. The Shapiro-Wilk test was used to verify the normality of the data. A two-sample t-test was used for the intergroup comparisons of the cephalometric measurement. Statistical analysis was performed by two of the co-authors (M.Y.H. and R.A).

## Results

Participant flow and follow-up

Twenty patients were enrolled (10 male, 10 female) in the RMI group at a mean age of 9.7±0.66 years, whereas 20 patients were enrolled (11 male, nine female) in the control group at a mean age of 9.9 ± 0.54 years. No patients were lost to follow-up (Table 2).

**Table 2:** Basic sample characteristics concerning age and gender for each group RMI: rapid molar intruder; SD: standard deviation; † Employing two-sample t-test; ‡ Employing chi-square test; NS: non-significant

Group	RMI	Control	P-value	RMI vs. Control
Age: years (mean ± SD)	9.7 ± 0.66	9.9 ± 0.54	0.266†	NS
Sex distribution: male/female	10/10	11/9	0.178‡	NS

The error in the method

The ICCs ranged from 0.967 to 0.999, which indicated high intra-examiner reliability for the measurement. Paired-sample t-tests showed that no systematic error was detected.

Cephalometric variables

The descriptive statistics of the linear and angular measurements made for both groups at the two assessment times are given in Table 3. Intragroup assessment of significant changes is also given in the same table, whereas inter-group differences of changes in these measurements are given in Table 4. The increase in the SNB in the RMI group was significantly greater than that of the control group (p<0.001). A statistically significant decrease was observed in the SN:GoMe angle and the sum of Bjork in the RMI group compared to the increase in the control group (p<0.001). The findings showed that the mean upper and lower first molar intrusion in the RMI group was 2.9 mm and 1.54 mm, respectively. At the same time, there was a slight mean extrusion of the upper and lower first molar in the control group (0.55 mm and 0.56 mm, respectively). There was a significant difference in the upper and lower molars' height between the RMI group and the control group (p<0.001). The overbite increased significantly in the RMI group compared to the control group (p<0.001). The lower lip-to-E line distance increased significantly in the RMI group compared to the slight decrease in the control group (p<0.001).

**Table 3:** Descriptive statistics of the angular and linear cephalometric measurements in each group and the P-values of significance testing for intra-group changes (i.e., before and after comparisons for each group) RMI: rapid molar intruder, SD: standard deviation; * highly significant difference (P < 0.001); † Employing paired-sample t test

Variable	RMI (n=20)					Control (n=20)				
	T1		T2		P-value	T1		T2		P-value†
	Mean	SD	Mean	SD		Mean	SD	Mean	SD	
SNA	79.62	2.25	79.87	1.90	0.114	79.96	2.03	80.09	1.75	0.363
SNB	74.56	2.30	76.35	2.24	<0.001***	75.55	2.51	75.77	2.55	0.073
ANB	5.17	1.47	3.47	1.29	<0.001***	4.43	1.30	4.32	1.48	0.542
N-S:GoMe	40.08	2.07	35.68	1.78	<0.001***	38.77	1.26	39.82	1.22	<0.001*
MM	33.35	3.85	29.26	3.07	<0.001***	31.14	2.09	32.38	2.06	<0.001*
Bjork Sum	402.30	3.99	398.00	3.40	<0.001***	400.51	3.04	401.93	3.13	<0.001*
Y axis	73.28	2.04	70.46	1.69	<0.001***	69.56	1.79	70.67	1.70	<0.001*
S-Go	67.35	3.80	68.53	3.89	<0.001***	67.63	2.47	67.84	2.46	0.071
N-Me	117.50	5.43	115.02	5.43	0.016*	112.20	5.11	113.21	5.11	<0.001*
U1-PP (mm)	28.31	2.07	28.44	2.14	0.285	25.64	1.94	25.75	1.97	0.110
U6-PP (mm)	22.17	1.68	19.27	1.63	<0.001***	19.18	1.91	19.73	1.94	<0.001*
L6-MP (mm)	39.20	2.19	37.66	2.16	<0.001***	37.42	1.77	37.98	1.76	<0.001*
U1:SN	103.85	4.08	103.90	4.13	0.143	106.70	2.89	107.29	2.88	0.053
U1:L1	113.31	6.41	112.49	6.26	<0.001***	114.20	4.23	113.30	5.07	<0.001*
Overbite	-2.59	1.19	1.85	1.06	<0.001***	-2.02	1.27	-2.21	1.33	0.209
Overjet	5.90	1.86	4.61	1.66	<0.001***	4.77	1.77	4.30	1.73	<0.001*
Li-E	2.55	0.55	3.12	0.59	<0.001***	2.96	0.78	2.84	0.85	0.230
Ls-E	1.25	0.72	2.05	0.71	<0.001***	1.35	0.66	1.70	0.69	0.263
Nasolabial	120.86	6.11	120.68	6.19	0.074	123.09	4.47	122.92	4.43	0.191
Labiomental	121.01	5.59	120.85	5.80	0.217	124.22	7.28	124.40	7.34	0.362

**Table 4:** Descriptive statistics of the changes observed in each group for the angular and linear measurements and the P-values of significance testing for inter-group differences concerning these changes RMI: rapid molar intruder, * p<0.05; ** p<0.001, † Employing two-sample t-test Refer to Table 1 for the definitions of the variables.

Variable	Control (n=20)	RMI (n=20)	Mean difference	P-value†
	T2-T1	T2-T1	Control - RMI	
	Mean	Mean		
SNA	0.13	0.25	-0.12	0.664
SNB	0.22	1.80	-1.58	<0.001**
ANB	-0.11	-1.70	1.59	<0.001**
N-S:GoMe	1.04	-4.40	5.44	<0.001**
MM	1.24	-4.09	5.34	<0.001**
Bjork Sum	1.42	-4.30	5.72	<0.001**
Y axis	1.10	-2.82	3.92	<0.001**
S-Go	0.21	1.18	-0.97	<0.001**
N-Me	1.02	-2.48	3.50	<0.001**
U1-PP (mm)	0.10	0.13	-0.03	0.860
U6-PP (mm)	0.55	-2.90	3.45	<0.001**
L6-MP (mm)	0.56	-1.54	2.10	<0.001**
U1:SN	0.59	0.05	0.54	0.635
U1:L1	-0.90	-0.81	-0.09	0.525
Overbite	-0.19	4.44	- 4.63	<0.001**
Overjet	-0.47	-1.29	0.82	<0.001**
Li-E	-0.12	0.57	-0.69	<0.001**
Ls-E	0.35	0.80	-0.45	0.043*
Nasolabial	- 0.17	- 0.18	0.01	0.950
Labiomental	0.18	-0.16	0.34	0.285

Harms

Spring fractures were observed in two cases in the RMI group, and the fractures were located at the end poles of the springs. These springs were replaced with new ones ensuring the same force levels.

## Discussion

AOB treatment is one of the most complicated cases in clinical practice, as the vertical dimension of the posterior alveolar heights must be controlled, and good patient cooperation should be obtained [17]. The current study was a randomized controlled trial designed to identify the changes caused by the treatment of skeletal anterior open bite in the mixed dentition using the rapid molar intruder on the dentofacial structures. Unlike most other studies which dealt with the AOB in mixed dentition, the present study was the first randomized controlled trial to evaluate the efficacy of RMI in correcting the early treatment of AOB. The control group was included in the study to identify the changes caused by growth without the intervention of orthodontic devices.

As for the skeletal changes, a statistically significant increase in the SNB angle occurred in the RMI group (= 1.80°) compared to the control group (= 0.22°). The observed increase in the SNB angle in the RMI group is in line with the study of Çinsar et al. (= 1.75°) [11] and the study of Hasan et al. (= 1.25°) [5]. This increase can be attributed to the anterior rotation of the mandible due to posterior teeth intrusion. The control group induced a modest increase in the SNB angle, which the spontaneous sagittal growth of the mandible could explain.

There was a statistically significant decrease in the mean value of the SN:GoMe angle (= 4.4°) and the mean value of the sum of Bjork (= 3.55°) in the RMI group compared to the increase in the control group (= 1.04° and = 1.40°, respectively). The decrease in the SN:GoMe angle and the sum of Bjork is in line with the study of Çinsar et al. (= 4.85° and = 4.55°, respectively) [11]. On the other hand, this decrease was larger than that found by Hasan et al. (= 1.61° and = 2.21°, respectively) [5] and Mousa et al. (= 1.44° and = 1.45°, respectively) [4]. This confirms the skeletal effect of the RMI in the vertical dimension.

As for the dentoalveolar changes, the overbite increased significantly in the RMI group (= 4.44 mm) compared to the control group (= 0.19 mm). The increase in the overbite in the RMI group was due to the counter-clockwise rotation of the mandible as a result of a reduction of the sum of Bjork, a reduction in the SN:GoMe angle, and posterior teeth intrusion. Similar results were found by Hasan et al. (= 4.54 mm) [5], Mousa et al. (= 4.91 mm) [4], and Çinsar et al. (= 4.5 mm) [11], where a fixed posterior bite block with tongue crib, an open bite Bionator (OBB), and a rapid molar intruder device, were used, respectively. On the other hand, the increase in overbite in the current trial was greater than what was reported by Turkkahraman and Cetin (= 3.79 mm) [8], Albogha et al. (= 3.1 mm) [6] and Iscan and Sarisoy (= 2.73 mm) [18], where a posterior bite block with high-pull headgear, an RMI with fixed posterior bite block, and a posterior bite block were used, respectively. However, the amount of the AOB in the previous studies was less than that of the current study.

There was a statistically significant intrusion of the upper and lower first molars (= 2.9 mm, = 1.54 mm, respectively) compared to the slight extrusion in the control group (= 0.55 mm, = 0.56 mm respectively). This intrusion of the upper and lower first molars in the RMI group was consistent with the findings of Çinsar et al. (= 2.54 mm and = 1.90 mm, respectively) [11] and Hasan et al. for the upper first molar only (= 1.21 mm) [5].

On the other hand, Albogha et al. [6] and Mebodi et al. [19] (using the posterior bite block) reported an average intrusion of the upper first molar of = 0.4 mm and = 0.5 mm, respectively, which was not statistically significant. This confirms the intrusion effect of the RMI in the vertical dimension of the posterior alveolar ridges.

The decrease in the overjet was statistically significant in the RMI group (= 1.29 mm) compared to the control group (= 0.47 mm). This finding was in agreement with the findings of Hasan et al. (= 1.25 mm) [5], Albogha et al. (= 1 mm) [6], and Çinsar et al. (= 1 mm) [11]. These findings can be attributed to anterior rotation of the mandible due to posterior teeth intrusion.

As for the soft tissue changes, there was a statistically significant lower lip retrusion in the RMI group (= 0.57 mm) compared to the slight lower lip protrusion (= 0.12 mm), which was due to the counter-clockwise rotation of the mandible. This lower lip retrusion in the RMI group is in line with the study of Çinsar et al. (= 0.85 mm) [11].

Limitations

There are some limitations of the present study. One is the relatively short follow-up period (i.e., nine months), and the failure to evaluate patient-reported outcomes measures (PROMs) is another limitation. A longer evaluation period is needed to assess possible relapse and the stability of the findings. Future similar work should consider pain, discomfort, and satisfaction levels when applying the rapid molar intruder.

## Conclusions

The rapid molar intruder was an effective appliance for the early treatment of the skeletal anterior open bite in children with mixed dentition. It provided favorable dentoalveolar, skeletal, and soft tissue changes compared to a control group of untreated patients.
